# Optimizing immune-related tumor response assessment: does reducing the number of lesions impact response assessment in melanoma patients treated with ipilimumab?

**DOI:** 10.1186/2051-1426-2-17

**Published:** 2014-06-18

**Authors:** Mizuki Nishino, Maria Gargano, Margaret Suda, Nikhil H Ramaiya, F Stephen Hodi

**Affiliations:** 1 Department of Radiology, Brigham and Women’s Hospital and Dana-Farber Cancer Institute, 450 Brookline Ave, Boston, MA 02215, USA; 2 Department of Medical Oncology and Department of Medicine, Dana-Farber Cancer Institute and Brigham and Women’s Hospital, 450 Brookline Ave, Boston, MA 02215, USA

**Keywords:** Immunotherapy, Tumor response, RECIST, Immune-related response criteria

## Abstract

**Background:**

Investigate the impact of the reduction of the number of target lesions on immune-related response assessment in advanced melanoma patients treated with ipilimumab.

**Method:**

Ninety patients (53 males, 37 females; age range: 25–87) with advanced melanoma treated with ipilimumab in two clinical trials were studied. Tumor measurements during trial allowing up to 5 lesions per organ and 10 lesions in total were retrospectively reviewed. A second set of tumor measurements allowing up to 2 lesions per organ and 5 lesions in total was generated. Immune-related response assessments by two measurements were compared.

**Results:**

The number of target lesions was significantly reduced when up to 2 per organ and 5 in total lesions were allowed (Wilcoxon P < 0.0001). The immune-related response assessment using reduced number of lesions was highly concordant with assessment using the original number of lesions (Spearman r for the percent change on 1^st^-3^rd^ follow-up: 0.860-0.970; κ_w_ for best immune-related response: 0.908). Median time-to-progression was 26.9 months (95%CI: 9.1-∞) by both assessments. Interobserver agreement of measurements was high for both assessments, with the concordance correlation coefficient above 0.98.

**Conclusion:**

Reduction of the number of target lesions did not significantly affect immune-related response assessment or the measurement variability in advanced melanoma patients treated with ipilimumab. Using up to 2 per organ and 5 in total target lesions is proposed to assess immune-related response, while it is important to keep other novel features of immune-related response criteria such as confirmation of progression and inclusion of new lesion measurements.

## Background

Anti-cancer immunotherapeutic agents have shown promising results in treatment of advanced cancer patients, as represented by survival benefit of ipilimumab in advanced melanoma patients [1-4]. Because of the distinct biologic mechanisms of anti-cancer activity of immunotherapeutic agents, cancer patients treated with immunotherapy need to be evaluated with special attention to the characteristics of immune-related tumor response. The immune related response criteria (irRC) has been developed to adequately characterize additional patterns of response and progression specific to patients treated with immunotherapy, that cannot be captured by the conventional criteria such as such as Response Evaluation Criteria in Solid Tumors (RECIST) [5,6]. The irRC has been increasingly recognized as one of the novel criteria to complement limitations of RECIST in patients treated with novel anti-cancer agents [5,6]. The irRC was recently used in a phase 2 clinical trial of ipilimumab in lung cancer patients to assess response and define trial endpoints [7].

We have previously compared immune-related response criteria using one-dimensional, longest diameters as in RECIST with the original irRC utilizing bidimensional measurements in advanced melanoma patients treated with ipilimumab. Unidimensional immune-related response assessment was highly concordant with bidimensional assessment, with better measurement reproducibility [8]. The use of the unidimensional measurements can be proposed, in order to reliably assess immune-related tumor response and provide results that can be directly compared to the results of trials based on unidimensional, RECIST-based assessment in the past decade.

RECIST, originally described in 2000, is the most commonly utilized criteria to assess tumor response to therapy in solid tumors, and is widely applied in clinical trials to determine endpoints, providing a basis for the approval of anti-cancer agents by Food and Drug Administration (FDA). The RECIST working group published a revised RECIST guideline (RECIST1.1) in January 2009, which has also been widely accepted and utilized to assess response and define progression in oncologic clinical trials [9-11]. One of the major changes of RECIST1.1 compared to the original RECIST (RECIST1.0) was the reduction of the number of target lesions, from 5 to 2 per organ, and from 10 to 5 in total. In our previous studies assessing the impact of RECIST1.1 guideline in response assessment of advanced non-small-cell lung patients, response assessment by RECIST1.1 was highly concordant with that by RECIST1.0 with improved reproducibility and required less time for measurements [12,13]. Other studies also described similar results in assessment of lung cancer and colon cancer, demonstrating the concordance of response assessment with decreased number of target lesions [14,15].

During the review of prospective tumor measurement records in our previous study comparing bidimensional versus unidimensional immune-related response assessment, it was noted that patients with advanced melanoma have multiple target lesions located in various organs and sites, not only in the visceral organs but also in the skin and muscles. The number of target lesions and the distribution of lesions in melanoma patients seemed distinct from previously studied cohorts such as patients with lung cancer or colon cancer. Given these observations, it was deemed worthwhile to study the immune-related response assessment by limiting the number of target lesions as defined in RECIST1.1, and compare the results with the assessment using the original number of target lesions.

The purpose of the present study was to evaluate the impact of the reduction of the number of target lesions in immune-related response assessment in advanced melanoma patients treated with ipilimumab. Given the results of our previous study comparing bidimensional versus unidimensional immune-related response assessment, unidimensional measurements were used for the purpose of the present study. We hypothesized that reducing the number of target lesions does not significantly affect the results of immune-related response assessment. We also evaluated the interobserver variability of measurements to assess the impact of the reduction of the number of target lesions on measurement variability.

## Results and discussion

### The number of target lesions and baseline measurements in 90 patients

**Figure 1:**
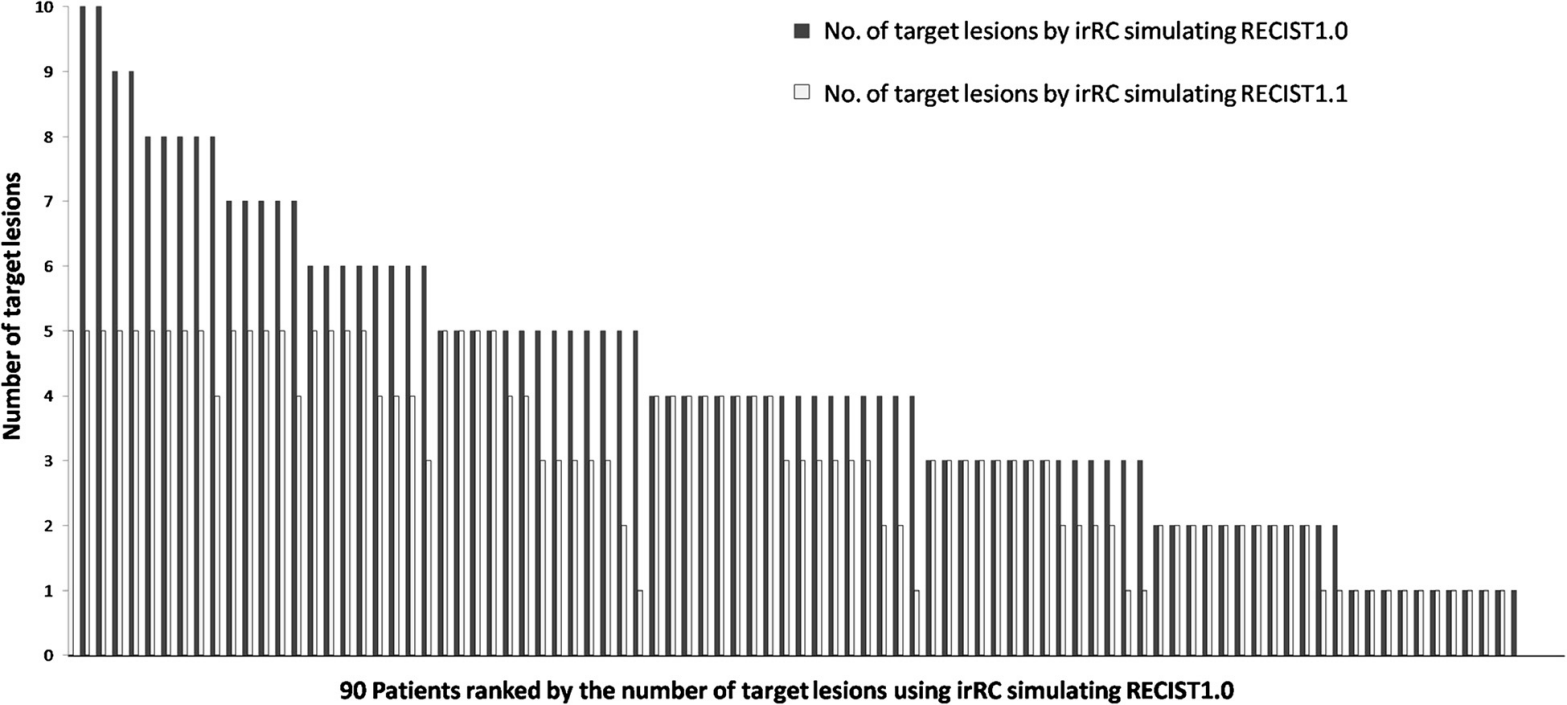
**The waterfall plot represents a number of target lesions for each patient using irRC simulating RECIST1.0 (dark gray bars) and irRC simulating RECIST1.1 (light gray bars).** The number of target lesions by irRC simulating RECIST1.1 was significantly smaller than that by irRC simulating RECIST1.0 (P < 0.0001).

**Figure 2:**
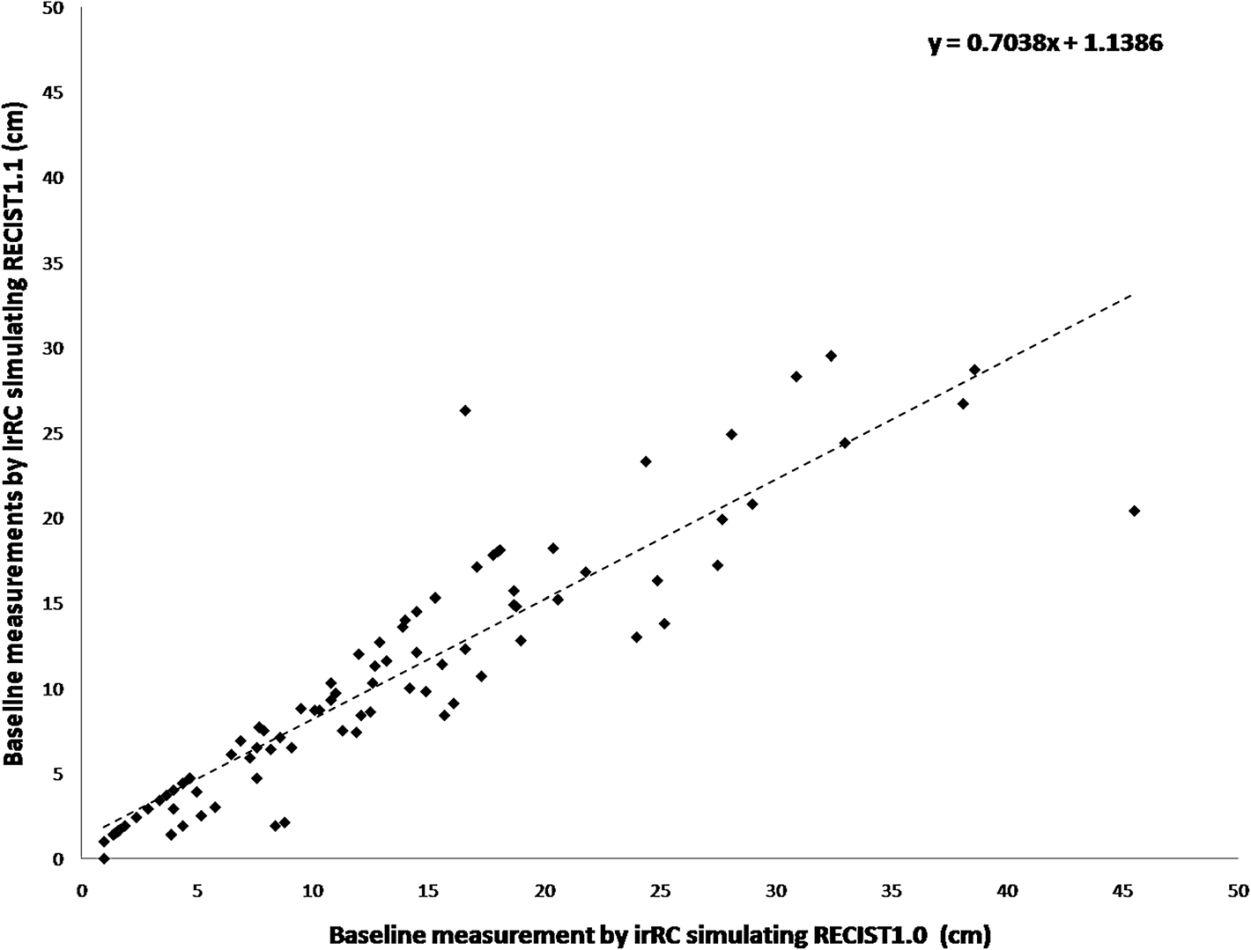
**The scatter plot represents the baseline measurements using irRC simulating RECIST1.0 and irRC simulating RECIST1.1.** A significant correlation was observed between baseline measurements using two criteria (Spearman correlation coefficient, 0.951 [95% CI: 0.93-0.97]).

The number of target lesions by irRC simulating RECIST1.1 was significantly smaller than that by irRC simulating RECIST1.0 (median number of target lesions per patient: 3 versus 4, respectively, P < 0.0001; total number of lesions in the cohort: 275 versus 381). The number of target lesions decreased in 50 of 90 patients (56%) when irRC simulating RECIST1.1 was used (median number of reduced target lesions: 1, range: 1–5) (Figure 1). The reasons of reduction of target lesions were a) reduction of maximum number of lesions per organ in 14 patients, b) criteria for measurable lymph nodes in 11 patients, c) reduction of maximum number of lesions in total in 7 patients, and d) combination of 2 or 3 of these reasons in 18 patients. One patient with one target lesion by irRC simulating RECIST1.0 had no target lesion when irRC simulating RECIST1.1 was used, because the lesion was a lymph node <15 mm in short axis.Baseline measurements by two criteria demonstrated a significant correlation, with a Spearman correlation coefficient of 0.951 (95% CI: 0.93-0.97) (Figure 2). The baseline measurements by irRC simulating RECIST1.1 was significantly smaller than that by irRC simulating RECIST1.0 (median: 9.0 versus 12.0 cm, respectively, P < 0.0001).

### Response assessment during immunotherapy by two criteria

**Figure 3:**
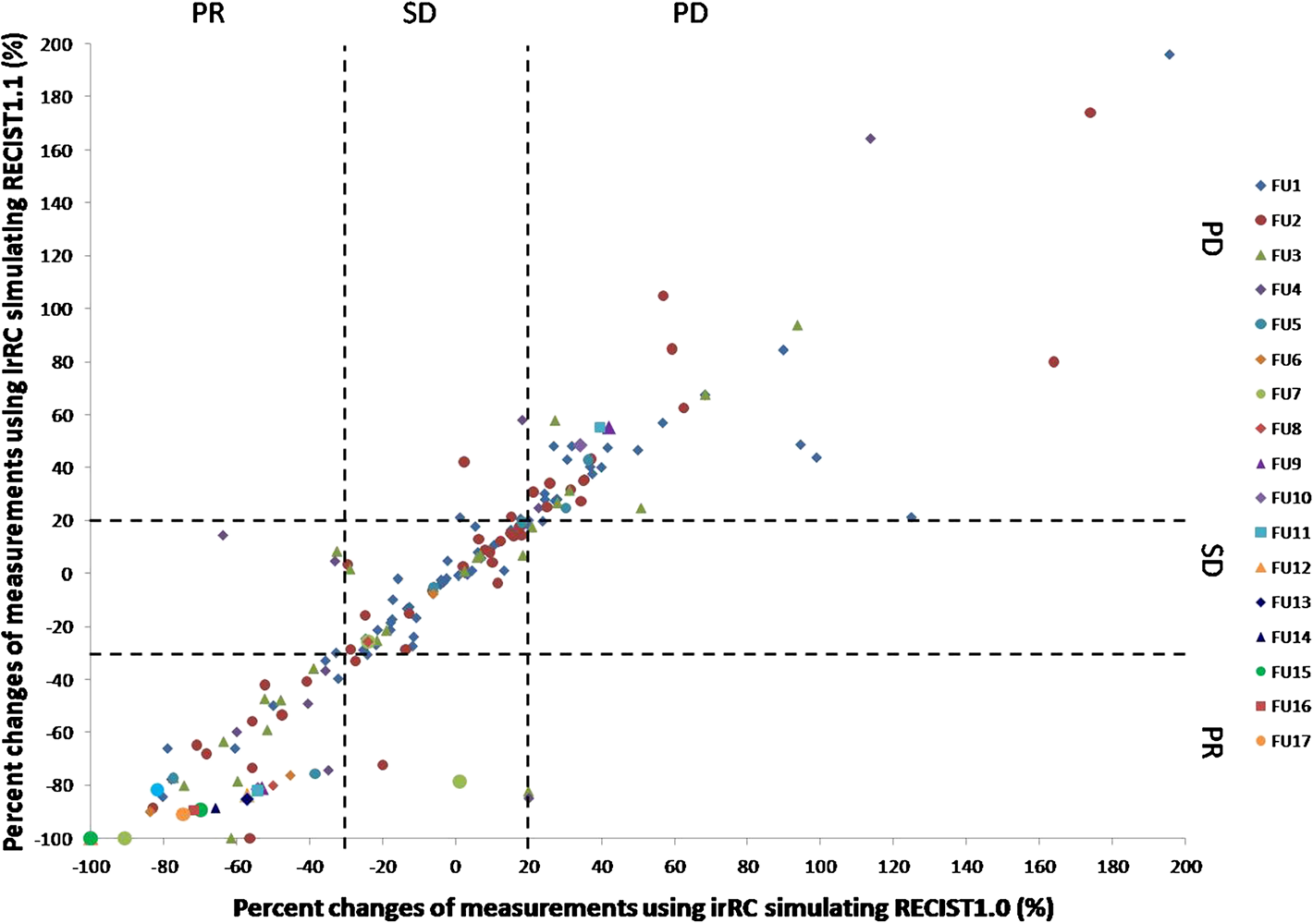
**The percent changes of measurements using irRC simulating RECIST1.0 and irRC simulating RECIST1.1 at each follow-up from the 1**^**st **^**to the 17**^**th **^**follow-up scans are shown.** Dashed lines at +20% and -30% represent the cut-off values for progressive disease and partial response, respectively. The observations within the lower left, middle center, and upper right boxes have concordant assessment between tow measurements, while observations in other boxes have discordant assessment. One concordant observation (+80% by irRC simulating RECIST1.0, +330% irRC simulating RECIST1.1) is not displayed since it is beyond the range of the Y axis.). The percent changes presented in the figure are in comparison with baseline measurements when tumors are decreasing to assess response, and in comparison with the nadir (the smallest measurement since baseline) when tumors are increasing to assess progression. These values are displayed since they are used to define response/progression in patients at the time of response assessment. Please also note that the number of patients decreases as the follow-up proceeds, starting from 71 patients at 1^st^ follow-up, 43 patients at the 2^nd^ follow-up, 27 patients at the 3^rd^ follow-up, and so on. There were 3 patients at the 12^th^ -14^th^ follow-up, 2 patients at 15^th^ and 16^th^ follow-up, and one follow-up at the 17^th^ follow-up.

**Figure 4:**
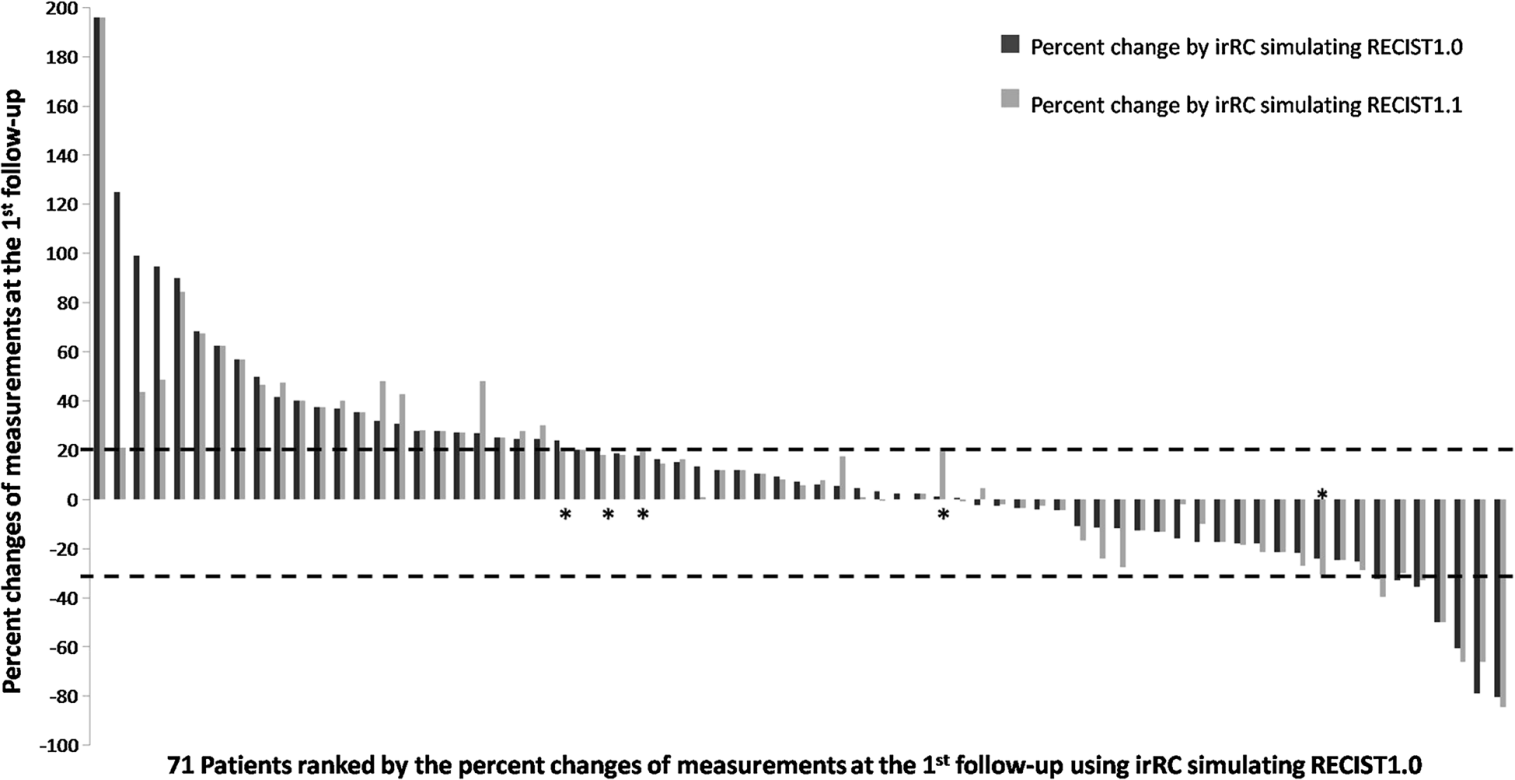
**The waterfall plot demonstrates the percent change of measurements by two criteria at the first follow-up scan.** Dark gray bars represent the percent changes by irRC simulating RECIST1.0, and light gray bars represent the percent changes by irRC simulating RECIST1.1. Dashed lines at +20% and -30% represent the cut-off values for progressive disease and partial response, respectively. 5 patients with discordant assessment are marked with asterisks (*).

**Figure 5:**
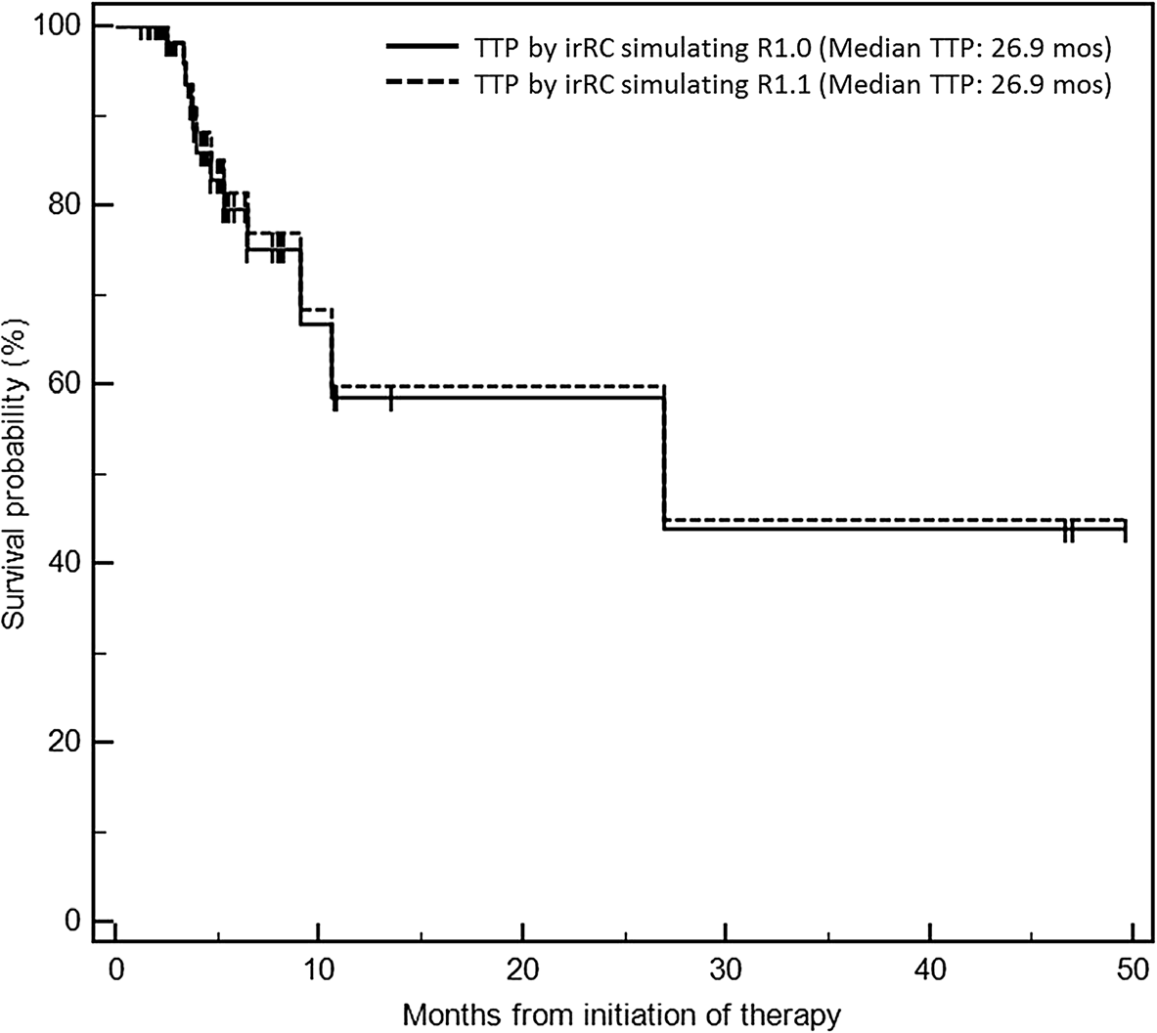
Time to progression by irRC simulating RECIST1.1 and irRC simulating RECIST1.0 had a median survival of 26.9 months (95% CI: 9.1-∞), without evidence of difference.

Response assessment was performed in 71 patients who had at least one follow-up CT during therapy among the 90 patients. Figure 3 demonstrates the percent changes of the measurements by two criteria at each follow-up scan (from 1^st^ to 17^th^ follow-up). High concordance was noted between the percent changes by two criteria, with Spearman correlation coefficient of 0.970 (95% CI: 0.95-0.98) for the first follow-up (n = 71), 0.946 (95% CI: 0.90-0.97) for the 2^nd^ follow-up (n = 43), 0.860 (95% CI: 0.71-0.94) for the 3^rd^ follow-up (n = 27). Figure 4 represents the waterfall plot of the percent changes by two criteria at the 1^st^ follow-up in 71 patients. Response assessment based on the percent changes of measurements by two criteria on the 1^st^-3^rd^ follow-up had almost perfect agreement, with κ_w_ of 0.913 (95% CI: 0.84-0.99) for the 1^st^, 0.901 (95% CI: 0.82-0.98) for the 2^nd^, and 0.837 (95% CI: 0.65-1.0) for the 3^rd^ follow-up. Best immune related response by two criteria demonstrated almost perfect agreement, with κ_w_ of 0.908 (95%CI: 0.82-0.99) (Table 1). Time to progression (TTP) had a median of 26.9 months (95% CI: 9.1-∞) by both criteria. Given the identical median TTP and its 95% confidence interval, there was no evidence of a difference in TTP by two criteria (Figure 5).

**Table 1:** Best overall response assessment by irRC simulating RECIST1.0 and irRC simulatin RECIST1.1 in 71 patients who had at least one follow-up CT

	**Assessment by irRC simulating RECIST1.0**				
**Assessment by irRC simulating RECIST1.1**	**irCR**	**irPR**	**irSD**	**irPD**	
irCR	1	1	0	0	1
irPR	0	8	1	0	10
irSD	0	0	52	1	53
irPD	0	0	1	6	7
	1	9	54	7	71

κ_w_ = 0.908 (95% CI: 0.82-0.99).

**Figure 6:**
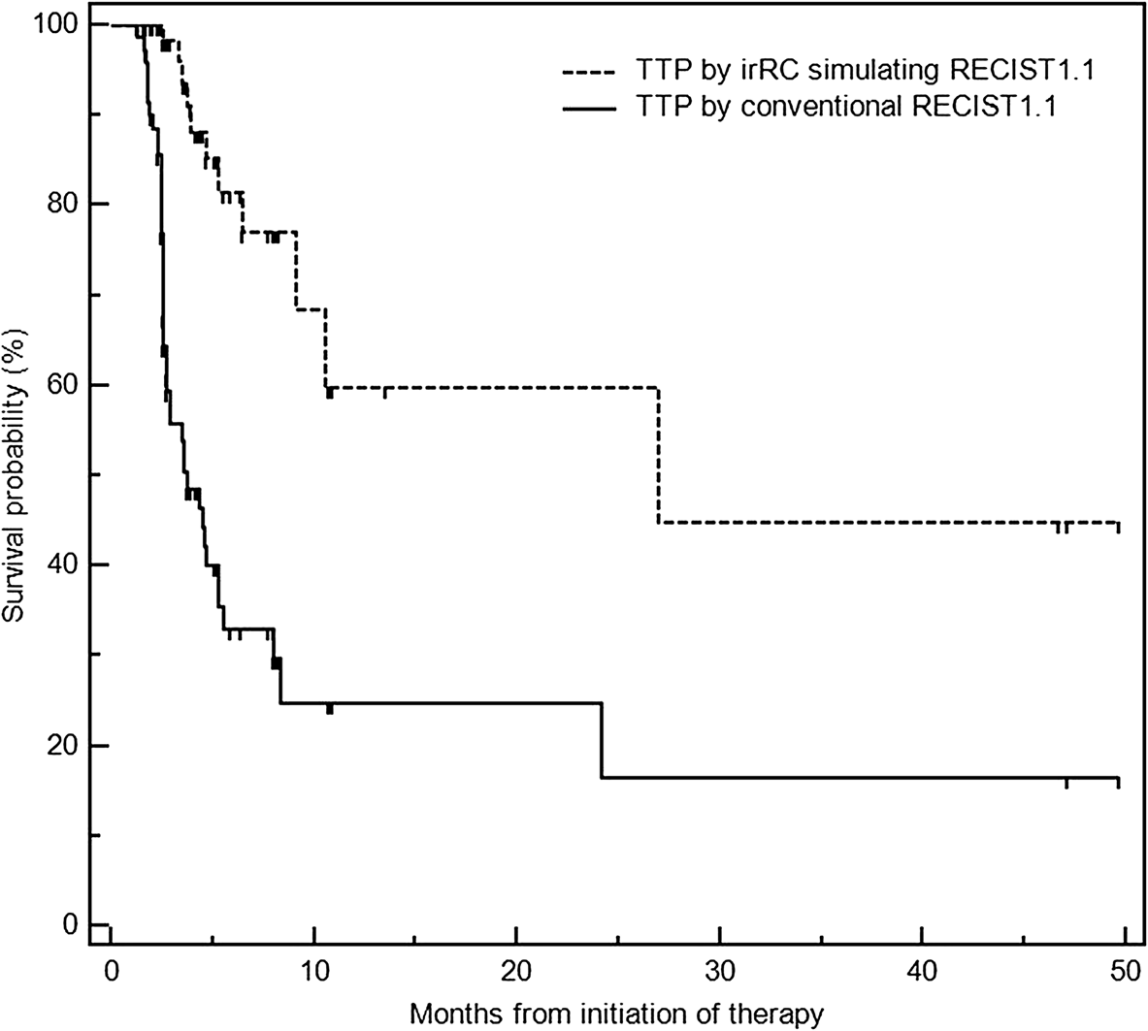
Time to progression by irRC simulating RECIST1.1 was much longer compared to that by conventional RECIST1.1 with median TTP of 3.7 mos (95% CI: 2.7-5.3).

In order to address the impact of irRC in tumor response assessment compared to the conventional assessment, the comparison was also made between the response assessment by irRC simulating RECIST1.1 and the assessment by the conventional RECIST1.1, which does not require confirmation of PD and defines PD at the time of appearance of a new lesion (rather than including the new lesion measurements in the tumor burden). TTP using the conventional RECIST1.1 was much shorter, with a median of 3.7 mos (95% CI: 2.7-5.3), compared to 26.9 mos using irRC simulating RECIST1.1 (Figure 6).

### New lesions

New measurable lesions were noted in 9 patients (number of new lesions: 4 in one, 2 in one, and 1 in seven patients, using irRC simulating RECIST1.0). In one patient with 4 new lesions by irRC simulating RECIST1.0, only 2 of the 4 new lesions were allowed by irRC simulating RECIST1.1 since all 4 lesions were peritoneal lesions. In a patient with one new lesion by irRC simulating RECIST1.0, the lesion did not qualify to be a measurable new lesion because it was a lymph node <15 mm in short axis. In another patient with one new lesion by both criteria, the lesion was a lymph node ≥15 mm in short axis, therefore, the measurement of the new lesion added to the sum was different between two criteria (the longest diameter versus short axis). For the remaining 6 patients, the number or the measurements of new lesions did not differ between the two criteria.

### Measurement variability

**Figure 7:**
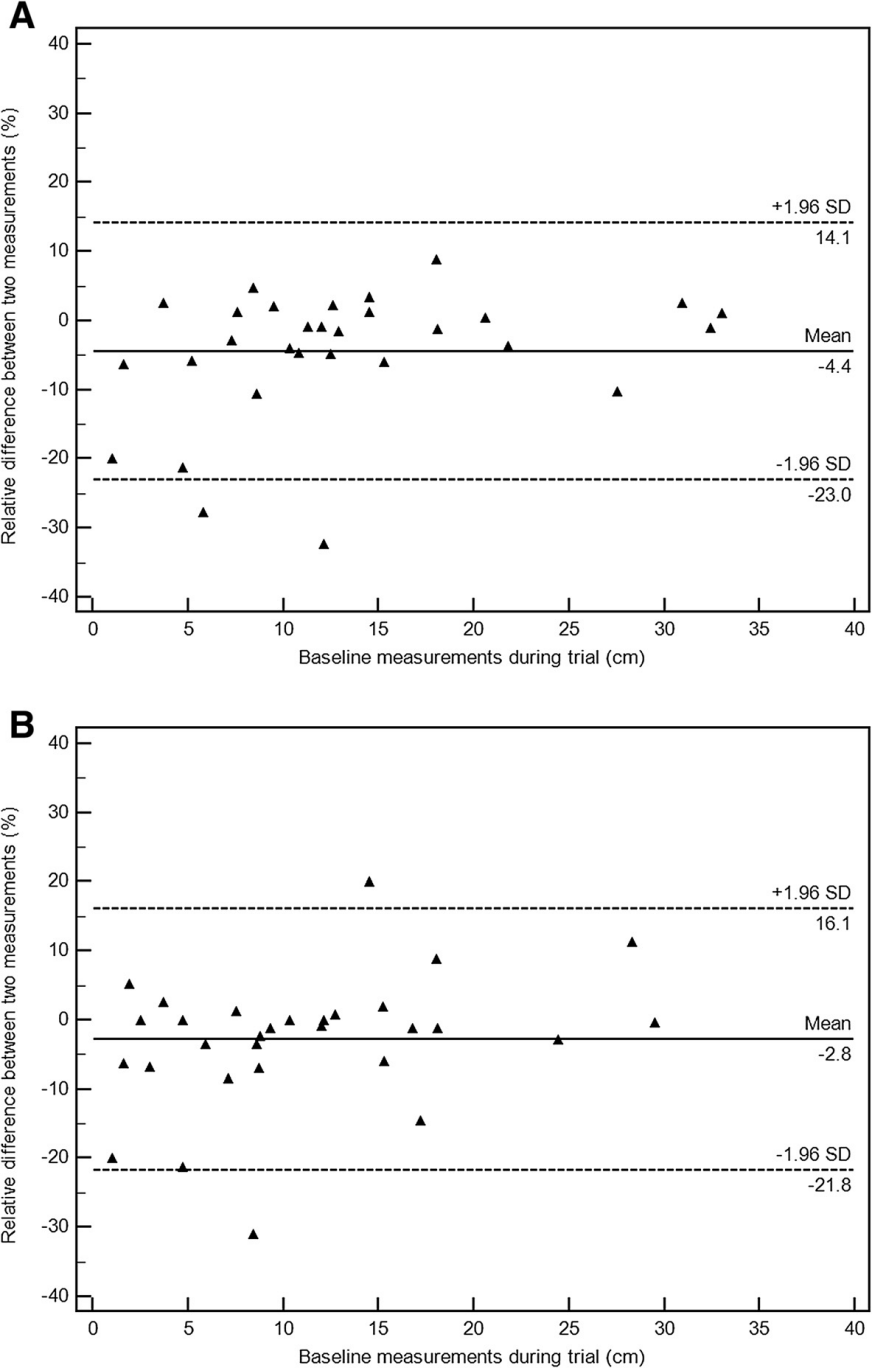
**Bland-Altman plots demonstrate the interobserver variability of measurements by irRC simulating RECIST1.0 (A), and by irRC simulating RECIST1.1 (B), on baseline scans in randomly selected 30 patients.** The 95% limits of agreement of measurements by irRC simulating RECIST1.0 were (-23.0%, 14.1%; Figure 7A, dashed lines), while the 95% limits of agreement of measurements by irRC simulating RECIST1.1 were (-21.8%, 16.1%; Figure 7B, dashed lines). The dotted lines represent the mean relative difference (%).

In randomly selected 30 patients, the concordance correlation coefficients (CCCs) between the measurements performed during the trial and the measurements by the radiologist performed in this study were 0.992 (95% CI: 0.98-0.99) for irRC simulating RECIST1.0, and 0.988 (95% CI: 0.98-0.99) for irRC simulating RECIST1.1. Figure 7 demonstrate Bland-Altman plots with 95% limits of agreement and the average relative difference. The 95% limits of agreement was (-23.0%, 14.1%) for irRC simulating RECIST1.0, and (-21.8%, 16.1%) for irRC simulating RECIST1.1.

## Discussion

The present study demonstrated that the immune-related response assessment using decreased number of target lesions simulating RECIST1.1 can provide highly concordant results compared to the assessment using the original number of target lesions according to RECIST1.0. The measurement variability was almost identical between two assessments. Based on the results, the number of target lesions in immune-related response assessment can be reduced to up to 2 per organ and up to 5 in total as defined in RECIST1.1, without significant changes in response assessment results and reproducibility of measurements.

Significant reduction of the number of target lesions were noted when irRC simulating RECIST1.1 was used, resulting in the reduction of the number of lesions in 56% of the patients. The result was expected since irRC simulating RECIST1.1 allows only up to 2 lesions per organ and up to 5 lesions in total, while irRC simulating RECIST1.0 allows up to 5 lesions per organ and 10 lesions in total, and advanced melanoma patients often had more than 2 lesions per organ and sometimes more than 5 lesions in total. The larger lesions based on unidimensional measurements remained as target lesions when the number of lesions exceeded the limit, since larger lesions were considered to represent tumor burden during the course of therapy. We also applied the revised criteria for measurability of lymph node (≥15 mm in short axis) as defined in RECIST1.1, which resulted in a reduction of the number of target lesions in a total of 21 patients (as a sole reason in 11 patients and in combination with other reasons in 10 patients). The revised lymph node guideline also resulted in a loss of target lesion in one patient whose only measurable lesion was a lymph node <15 mm in short axis, indicating the guideline can affect the trial eligibility of patients since many trials require at least one measurable lesion for enrollment, and further studies are needed to address this important clinical issue. The observation is concordant with the results in other studies comparing RECIST1.0 and RECIST1.1 in other solid tumors such as lung cancer and prostate cancer [16]. It has been shown that RECIST1.1 with decreased number of lesions requires significantly less time compared to RECIST1.0, which will to lead time saving and workflow in clinical practice [13].

The percent changes of measurements at each follow-up were highly concordant between the two criteria, and the response assessment results for the first three follow-up studies had almost perfect agreement, with the weighted kappa value >0.837, in spite of the significant reduction of the number of target lesions by irRC simulating RECIST1.1. The best immune-related response was also highly concordant between the two criteria, with weighted kappa value of 0.908. These results were concordant with our hypothesis, that reducing the number of target lesions does not significantly affect the results of immune-related response assessment. The results were also quite similar to the previous studies comparing RECIST1.0 and RECIST1.1 in advanced lung cancer patients [12-14]. In a study from our group evaluating RECIST1.0 and 1.1 in advanced NSCLC patients treated with erlotinib, the response assessment results were highly concordant between the two criteria with weighted κ of 0.905, while the number of target lesions was significantly reduced by RECIST1.1 [12]. Similar observation was also noted in the genomically defined cohort of NSCLC patients with sensitizing EGFR mutations treated with EGFR inhibitors, which demonstrated measurements using RECIST1.1 require less time compared to measurements using RECIST1.0 [13].

Time to progression analysis resulted in identical median TTP and its 95% CI, further supporting our hypothesis. The result of TTP is concordant with the prior study of NSCLC patients, which demonstrated no significant difference in TTP by RECIST1.0 and RECIST1.1 [12]. Confirmation by 2 consecutive studies was required for both criteria, since this was one of the most important feature of the original irRC to differentiate initial apparent increase of tumor due to accumulation of immune cells from true progression. The cut off values to define response and progression were -30% and +20% as defined by RECIST1.0 and 1.1, because the assessments in the present study were based on unidimensional measurements. Although the original irRC published in 2009 used bidimensional measurements based on WHO criteria, we chose to utilize unidimensional, longest diameter measurements in this study. This was because 1) the longest diameter measurements based on RECIST were used in assessing response and defining trial endpoints in the majority of the clinical trials of solid tumors in the past decade, and 2) our previous study demonstrated that unidimensional measurements provide immune-related response assessment which is highly concordant with the assessment using bidimensional measurements with better reproducibility [8]. TTP using irRC simulating RECIST1.1 was much longer compared to TTP using the conventional RECIST; this is because 1) the requirement of confirmatory observation for PD by irRC allows patients to continue therapy beyond initial increase of tumor burden without being assessed as having “progression” immediately at the first scan showing ≥20% increase, and 2) new lesions by irRC does not define PD (and instead their measurements are included in the sum of the tumor burden) and therefore patients may continue therapy without being labelled as “progressors” even in the presence of new lesions. The results are indicative of the impact of these 2 unique features of irRC on assessing response and defining trial endpoints, which should be kept in mind when designing trials of immunotherapeutic agents and defining trial endpoints, and advocate the need for future studies.

Particular attention was paid in the study design as to the assessment of new lesions and their measurements. The irRC simulating RECIST1.0 allows up to 5 new lesions per organ and up to 10 new lesions, while irRC simulating RECIST1.1 allows up to 2 new lesions per organ and up to 5 new lesions in total; this is regardless of the number of target lesions at baseline. Both criteria included the measurements of new lesions in the sum of the measurements to define response and progression, since this was one of the major features of immune-related response assessment. While some differences were noted between the two criteria in 3 of the nine patients with new lesions (the difference was in the number of new lesions allowed in one patient, in the measurability of a new lymph node in one patient, and in the measurement of a new lymph node in another patient), no difference was noted in best response assessment and TTP in these 9 patients by two criteria. Since the appearance of new lesion defines progression in RECIST, there is no guideline as the how many new lesions are allowed by RECIST. We based the upper limits of new lesions on the number of target lesions allowed per organ and in total by RECIST1.0 and 1.1, given that the original irRC defines the limits of number of new lesions using the limits for target lesions (up to 5 per organ, 5 cutaneous lesions and 10 visceral lesions) [5]. In the present study, cutaneous lesions were counted toward in the total number of target lesions.

We assessed interobserver agreement of the measurements on baseline scans by two criteria, since the measurement variability is an important factor when developing and optimizing tumor response criteria. Interobserver agreement of measurements was very high for both criteria, with CCC above 0.98. The 95% limits of agreement were very similar between the two criteria, (-23.0%, 14.1%) for irRC simulating RECIST1.0, and (-21.8%, 16.1%) for irRC simulating RECIST1.1. The result is somewhat different from the prior study of advanced NSCLC patients, where measurements using RECIST1.1 was more reproducible than measurements using RECIST1.0 [12]. The reason for the difference may be due to the difference of the cohort with different tumor types, while the number of patients studied in each study is small to identify definitive reasons. Nevertheless, it is reassuring that the measurements with decreased number of lesions were as reproducible as measurements with the original number of lesions. More important observation is that the threshold used to define response and progression, -30% and +20%, was beyond the range of the 95% limits of agreement and therefore can be considered to represent true change of tumor burden rather than the measurement error [17,18]. The result provide supporting evidence of the cut-off values used in RECIST, while one should note that 20% change for progression can be within the upper limits depending on the way we subtract one measurement from the other to obtain difference (i.e., the 95% limits of (-23.0%, 14.1%) can be (-14.1%, 23%) if measurement 1 was subtracted from measurement 1 in the method). This is also consistent with the prior studies of measurement variability, which described the risk of misclassifying patients as progressors [17-19]. However, such risk of misclassification for progression is probably less in irRC since it requires confirmation on two consecutive scans for progression, which is not required by RECIST [5,9-11].

The limitations of the study include retrospective design with a cohort of patients treated at a single institution. The results of the study can be validated in a larger multicenter cohort of melanoma patients treated with immunotherapy. Along with the validation study, it is necessary to investigate the association between response assessment by irRC and overall survival, to find out which of the criteria most accurately predicts survival. While either of two criteria compared in the study were not fully established as guideline to assess immune-response, we defined the details of the two criteria based on RECIST1.0 and RECIST1.1 while keeping the important novel features of irRC published in 2009. Each of the criteria details used in the study has its own rationale as described in the Method and Discussion, and made by consensus of experienced oncoradiologists who have focused on tumor response assessment. The results of the study should be interpreted in the context of the study design.

## Conclusions

In conclusion, reducing the number of target lesions did not significantly affect the results of immune-related response assessment or the measurement reproducibility in advanced melanoma patients treated with ipilimumab. These results are indicative that utilize up to 2 per organ and 5 in total target lesions may be sufficient for immune-related response assessment, as long as the important features of irRC such as confirmation of progression and inclusion of new lesion measurements are kept, while these observations need to be validated in a larger trial. Decreasing the number of target lesions may help to increase the practicality of immune-related response assessment, and help to make irRC more widely applicable and adoptable for radiologists who are primarily using RECIST 1.1 criteria.

## Methods

### Patients

The study population consisted of 90 patients (53 males, 37 females; median age 62, range 25–87 years) with advanced melanoma treated with ipilimumab at the Dana-Farber Cancer Institute in two prospective clinical trials, whose prospective tumor measurement records during trials are available for review. Seventy-five patients were treated in a phase 2, multicenter treatment protocol for expanded access of ipilimumab (BMS-734016) monotherapy in subjects with histologically confirmed unresectable stage III or stage IV melanoma, and 15 patients were treated in a phase 1 trial of Anti-Cytotoxic T-Lymphocyte-Associated Antigen-4 (anti-CTLA-4) Humanized Monoclonal Antibody (ipilimumab) [20,21]. Fourteen patients in the phase II trial received a dose of 10 mg/kg of ipilimumab, while the remaining 76 patients received a dose of 3 mg/kg. The protocols were approved by the Dana-Farber/Harvard Cancer Center institutional review board, and all patients provided written informed consent.

### Tumor response assessment

The original tumor measurements were performed prospectively during the trial by staff radiologists at Dana-Farber Cancer Institute at the baseline and at every follow-up computed tomography (CT) examinations, allowing up to 5 lesions per organ, and up to 10 lesions in total according to RECIST1.0 [9]. CT scans were performed at every 12 weeks in principle, while shorter interval follow-up (i.e., 4 weeks) were performed if necessary for the purposes such as confirmation of response or progression [8]. The tumor measurement records included the number of the treatment cycle, date of assessment, imaging modality, target lesion description and bidimensional measurements, descriptions of nontarget lesions, the presence or absence of new lesions with measurements if present.

The original tumor measurement records completed in the trials were retrospectively reviewed by a board-certified radiologist (M.N.) with 8 years of experience in oncologic imaging, and two sets of immune-related tumor response assessments were performed (Table 2). The first assessment utilized irRC simulating RECIST1.0, allows up to 5 lesions per organ, up to 10 lesions for target lesions. The longest diameter measurements were used for all target lesions. The longest diameters of new lesions, if any, were also included in the measurements, since this is one of the important characteristics of irRC. Up to 5 new lesions per organ, up to 10 new lesions were allowed. The sum of the longest diameters of all target lesions (and new lesions, if any) was calculated at baseline and each follow-up study.

**Table 2:** Summary of measurement approaches for two assessments

	**irRC simulating RECIST1.0**	**irRC simulating RECIST1.1**
Number of target lesions	Up to 5 per organ, up to 10 in total	Up to 2 per organ, up to 5 in total
Measurable lesions	≥10 mm in the longest diameter for all lesions	≥10 mm in the longest diameter for all lesions except for lymph nodes
		≥15 mm in short axis for nodes
Measurement of each lesion	The longest diameter for all target lesions	The longest diameter for non-nodal lesions, short axis for lymph nodes
New lesions	The presence of new lesion does not define progression	Same as irRC simulating RECIST1.0 except:
	The measurements of the new lesion (s) are included in the sum of the measurements	A lymph node has to be ≥15 mm in short axis to be a measurable new lesion and its short axis measurement is included in the sum
	Up to 5 new lesions per organ, up to 10 new lesions in total can be added to measurements	Up to 2 new lesions per organ, up to 5 new lesions in total can be added to the measurements

The second set of assessment utilized irRC simulating RECIST1.1, which included the revised guidelines for the number of target lesions and the measurement of lymph nodes described in RECIST1.1 [10] (Table 2). The criteria allowed up to 2 lesions per organ and up to 5 lesions in total, and required at least 15 mm in short axis for lymph nodes to be measurable [10]. In this assessment by irRC simulating RECIST1.1, when the number of target lesions exceeded the limits (up to 2 per organ and 5 in total), larger lesions based on the unidimensional measurements (the longest diameter for non-nodal lesions and the short axis for lymph nodes) were selected to remain as target lesions. Lymph nodes less than 15 mm in the short axis were excluded from target lesions. The short-axis measurements were used for lymph nodes instead of the longest diameters [11]. Up to 2 new lesions per organ, up to 5 new lesions in total were allowed, and the measurements of new lesions were included in the sum of the measurements. New lymph nodes have to be ≥15 mm in short axis to be measurable, and the short axis measurements were included in the sum for new nodes. Although the second assessment utilized irRC simulating RECIST1.1, new lesion by FDG-PET was not included in the response assessment since new lesions do not define progression by the original irRC [5]. Absolute ≥5 mm increase (in addition to ≥20%) for progression by RECIST1.1 was not used, because 1) the main purpose was to evaluate the impact of reducing the number of target lesions, and 2) the 5 mm requirement affected only a minority of patients in our prior study of advanced NSCLC patients [13].

The sum of the measurements of target lesions (and new lesions, if any) at baseline and each follow-up study, and the percent changes at each follow-up were calculated for the two sets of assessments. Response assessment was assigned at each follow-up using the cut-off values for percent changes used in RECIST1.0 and 1.1 (≥20% increase for disease progression (PD), ≥30% decrease for partial response (PR)), since both assessments used unidimensional measurements as in RECIST [9-11]. Complete response (CR) required disappearance of all lesions including lymph nodes for irRC simulating RECIST1.0, while all lymph nodes must be < 10 mm short axis by irRC simulating RECIST 1.1 [9-11]. Confirmation by two consecutive observations not less than 4 weeks apart was required for irCR, irPR and irPD, since it is one of the most important features of the original irRC to capture immune-related response [5]. Best overall response was assigned to each patient using the two criteria. Best overall response is the best response recorded from the start of the study treatment until the end of treatment or the last follow-up, taking into account any requirement for confirmation.

### Measurement variability

To assess variability of measurements, a board-certified radiologist (M.N.) performed tumor measurements of target lesions on baseline scans in a randomly selected 30 patients. The prospective baseline tumor measurements during trials in these patients were performed by staff radiologists at our institution other than the radiologist (M.N.). The radiologist performed measurements of the target lesions that have been already selected during trials. Tumor table templates indicating the location, description, and series and image numbers of target lesions (such as “right lower lobe lung lesion, series 3, image 30”) were provided to the radiologist, who was not allowed to access the original measurements during the trials. Measurements were performed using a measurement tool on a picture archiving communication system (PACS) workstation (Centricity, GE Healthcare), which was also used for measurements during the trials. The sum of the longest diameters of all target lesions was recorded for the measurements by irRC simulating RECIST1.0. The measurements by irRC simulating RECIST1.1included the lesions that remained as target after applying revised guidelines for the number of lesions and lymph node measurability, and the sum of the longest diameters of non-nodal target lesions and the short axis measurements of target lymph nodes were recorded.

### Statistical analysis

The number of target lesions by two assessments was compared using a Wilcoxon signed rank test. The baseline measurements and the percentage changes at each follow-up by two assessments were compared using Spearman correlation. A weighted kappa analysis was performed to assess the level of agreement between responses by the two assessments using Fleiss-Cohen quadratic weights [13]. Agreement between the two assessments was categorized as poor (weighted κ < 0), slight (weighted κ = 0–0.20), fair (weighted κ = 0.21–0.40), moderate (weighted κ =0.41–0.60), substantial (weighted κ = 0.61–0.80), and almost perfect (weighted κ > 0.80) [12,13]. Time to progression (TTP) according to two measurement records was estimated using the Kaplan-Meier method [22].

Interobserver variability was assessed using CCCs, mean relative difference (%), and 95% limits of agreement (%), for the measurement using irRC simulating RECIST 1.0 and the measurements using irRC simulating RECIST1.1. CCC was used to assess reproducibility of two measurements, as described previously [17,18]. Assuming two measurements have mean u_1_ and u_2_, with variance $\sigma_{1}^{2},\sigma_{2}^{2},$ and covariance σ_12,_$\mathrm{CCC}=\left(2,\sigma_{2}\right)/\left[\sigma_{1}^{2},+,\sigma_{2}^{2},+,{\left(u_{1-},u_{2}\right)}^{2}\right].$ CCCs are composed of a measure of precision (how far each pair of measurements deviates from the best-fit line through the data) and a measure of accuracy (the distance between the best-fit line and the 45 line through the origin). A value of 1 indicates perfect agreement and -1 indicates perfect reversed agreement [23]. Bland-Altman plots were used to demonstrate agreement in the two measurements, with 95% limits of agreement and the average relative difference, computing the mean relative difference (%) between the two measurements (100*[M_1_-M_2_]/M_1_; M_1_ = Measurements during trial, M2 = Measurements by the radiologist in this study) [18].

All *p* values are based on a two-sided hypothesis. A *p* value of less than 0.05 was considered to be significant.

## Abbreviations

irRC: Immune related response criteria; RECIST: Response Evaluation Criteria in Solid Tumors; FDA: Food and Drug Administration; anti-CTLA-4: anti-Cytotoxic T-Lymphocyte-Associated Antigen-4; CT: Computed tomography; PACS: Picture archiving communication system; PD: Disease progression; PR: Partial response; CR: Complete response; TTP: Time to progression; CCCs: Concordance correlation coefficients.

## Competing interests

Nishino, Ramaiya, Gargano, Suda: None. Hodi: served as a non-paid consultant to Bristol-Myers Squibb; received clinical trial support from Bristol-Myers Squibb.

## Author’s contributions

MN: Conception and study design, data acquisition by performing tumor measurements and review of the images and measurements, statistical analysis and interpretation of data, drafting and revising the manuscript. NHR: Conception and study design, interpretation of data, drafting and revising the manuscript. MG: Data acquisition by medical record review, drafting and revising the manuscript. MS: data acquisition by medical record review, drafting and revising the manuscript. FSH: Conception and study design, interpretation of data, drafting and revising the manuscript. All authors read and approved the final manuscript.
